# Supplementary material for: ReorientExpress: reference-free orientation of nanopore cDNA reads with deep learning
Source: Genome Biol. 2019 Nov 29;20:260. doi: 10.1186/s13059-019-1884-z (PMC6883653; doi:10.1186/s13059-019-1884-z)
Supplement: Supplementary file 1 — Additional file 1. Additional figures and tables referenced in the article. [file 13059_2019_1884_MOESM1_ESM.pdf]

Additional File 1

ReorientExpress: reference-free orientation of nanopore cDNA reads with deep learning

Angel Ruiz-Reche[1,#], Akanksha Srivastava[2,3,#], Joel A. Indi[1,4], Ivan de la Rubia[1], Eduardo Eyras[2,3,5,*]

[1]Pompeu Fabra University, E08003, Barcelona, Spain
[2]The John Curtin School of Medical, Australian National University, Acton ACT 2601, Canberra, Australia
[3]EMBL Australia Partner Laboratory Network and the Australian National University, Acton ACT 2601, Canberra, Australia
[4]Instituto de Medicina Molecular, Faculdade de Medicina, Universidade de Lisboa, Lisbon, Portugal
[5]IMIM - Hospital del Mar Medical Research Institute. E08003 Barcelona, Spain.

[#] these authors contributed equally
* correspondence to eduardo.eyras@anu.edu.au

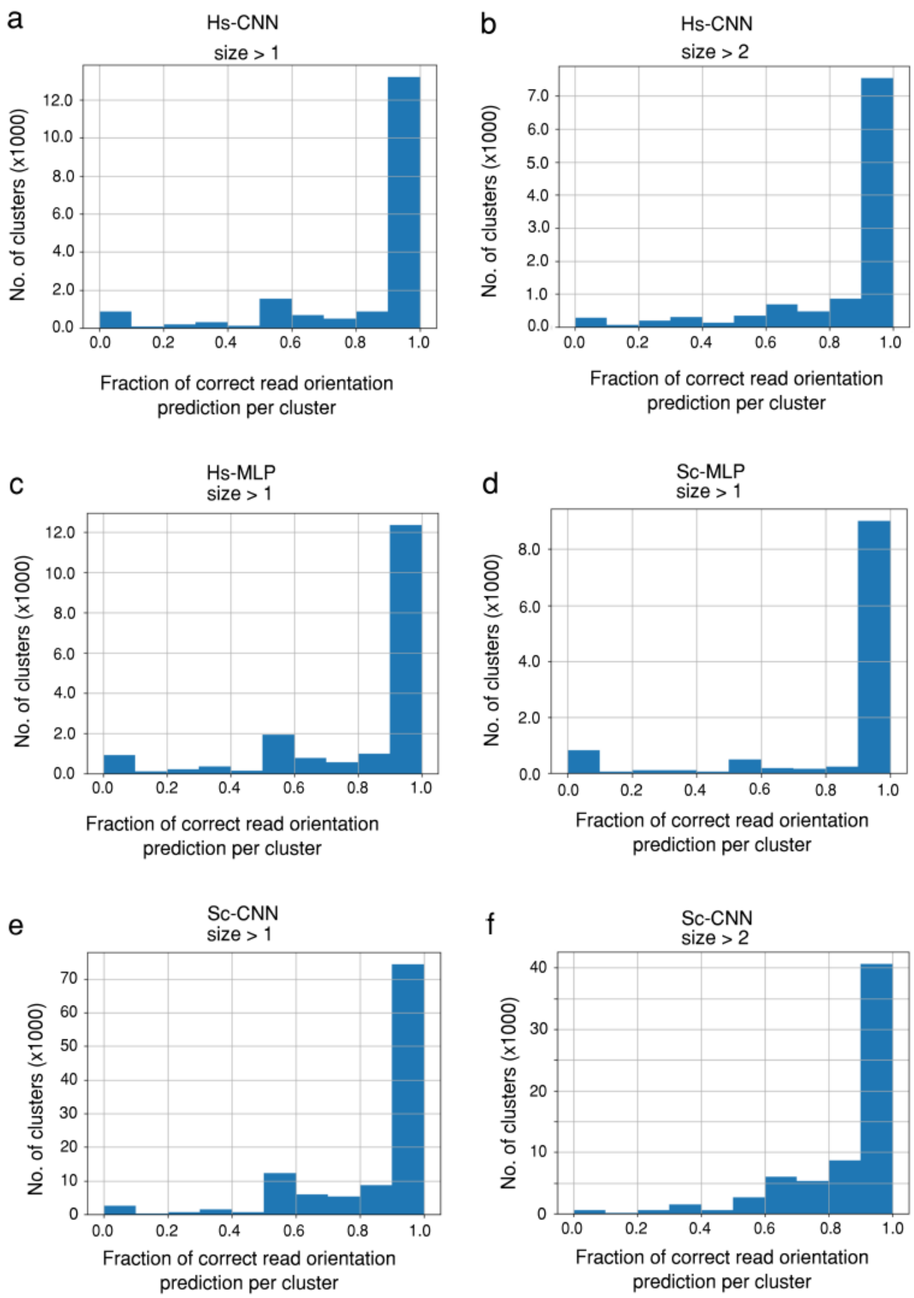

Figure 1. Proportion of corrected reads per cluster. Number of clusters (y axis) according to the proportion of ONT cDNA reads in the cluster with orientation correctly predicted by ReorientExpress (x axis) for the CNN model trained on the human transcriptome (Hs-CNN) for clusters with >1 reads **(a)** and for clusters with >2 reads **(b)**, for the MLP model trained on the human transcriptome (Hs-CNN) for clusters with >1 reads **(c)**, for the MLP model trained on the S. cerevisiae transcript (Sc-MLP) for clusters with >1 reads **(d)**, and for the CNN model trained on the S. cerevisiae transcriptome (Sc-CNN) for clusters with >1 reads **(e)** and for clusters with >2 reads **(f).** The number of clusters with more than 50% of their reads correctly oriented are given in Table 5. The total number of reads correctly oriented after using a majority vote are given in Table 6.

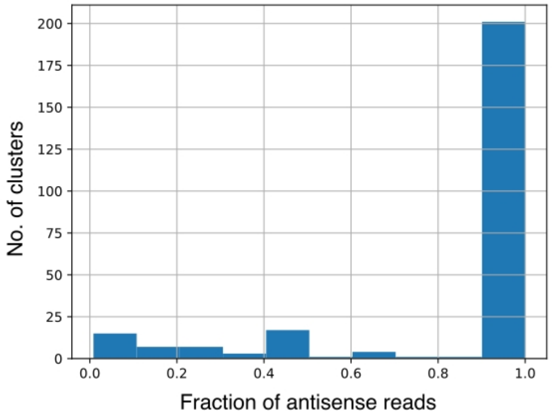

Figure 2. Clusters with antisense cDNA reads. The plot shows the clusters that contained 1 or more reads labelled as antisense, according to the proportion of reads in each cluster that were of type antisense. The plot shows that the majority of clusters with antisense reads consists of 100% antisense reads. The clusters that have 100% of their reads labeled as antisense are 201 (78%), which correspond to 882 antisense reads from the total of 976 antisense reads, i.e. 90%.

Layer	Type	Activation	Nodes	Dropout Rate
0	Input	-	1364	
1	Dense	ReLu	500	
	Dropout	-		0.3
2	Dense	ReLu	250	
	Dropout	-		0.3
3	Dense	ReLu	125	
	Dropout	-		0.3
4	Dense	ReLu	62	
	Dropout	-		0.3
5	Dense	ReLu	31	
	Dropout	-		0.3
6	Dense	Sigmoid	1	

Table 1. Architecture of the MLP model. The MLP model has 5 hidden layers, with the last layer providing the probability that a read is not in the correct orientation, and with dropout layers to reduce overfitting. The first layer is the input, with as many nodes as the number of normalized frequencies. For k-mers with k=1,...,5, there are 4 + 16 + 64 + 256 + 1024 = 1364 frequencies. For every layer (column Layer), the activation function (Activation) and the number of nodes (Nodes) is described. The activation functions used are the Rectified Linear Unit (ReLu) and the sigmoid. For each layer, the Dropout layer inserted immediately after the dense layer and the dropout rate is indicated.

Layer	Type	Activation	No. of filters	Filter size/nodes	Dropout Rate
1	Conv1	ReLu	32	11 x 4	-
	Pool1	-	32	4 x1	-
2	Conv2	ReLu	64	3 x 1	-
	Pool2	-	64	2 x1	-
3	Conv3	ReLu	96	3 x 1	-
	Pool3	-	96	2 x1	-
4	Flatten	-	-	-	-
5	Dense	ReLu	-	256	-
	Dropout	-	-	-	0.4
6	Dense	ReLu	-	256	-
7	Dense	softmax	-	2	-

Table 2. Architecture of the CNN model. The architecture consists of 3 convolutional layers (Conv1-3), 3 pooling layers (Pool1-3) and 3 dense layers, with different filter sizes. The Conv and Pool layers identify the important features and the dense layers combine these features together to make the prediction. The activation function used are the Rectified Linear Unit (ReLu) and the softmax. The dropout of 0.4 is used between first and second dense layer.

			Forward				Reverse				Average			
Model	Training	Testing	Prec.	Recall	F1	Reads	Prec.	Recall	F1	Reads	Prec.	Recall	F1	Reads
MLP	Human transcriptome	human cDNA reads	0.76	0.84	0.80	20655	0.89	0.82	0.85	30450	0.84	0.83	0.83	51105
MLP	Human transcriptome	Human DRS reads	0.86	0.86	0.86	25081	0.86	0.86	0.86	24919	0.86	0.86	0.86	50000
MLP	S. cerevisiae transcriptome	S. cerevisiae cDNA reads	0.91	0.93	0.92	19962	0.95	0.94	0.94	30460	0.93	0.93	0.93	50422
MLP	S. cerevisiae transcriptome	S. cerevisiae DRS reads	0.85	0.94	0.89	25023	0.93	0.83	0.88	24977	0.89	0.88	0.88	50000
CNN	Human transcriptome	human cDNA reads	0.79	0.90	0.84	21476	0.92	0.82	0.86	28525	0.86	0.86	0.85	50001
CNN	Human transcriptome	Human DRS reads	0.91	0.91	0.91	50000	0.91	0.91	0.91	50000	0.91	0.91	0.91	50000
CNN	S. cerevisiae transcriptome	S. cerevisiae cDNA reads	0.86	0.91	0.88	23095	0.92	0.87	0.90	26906	0.89	0.89	0.89	50001
CNN	S. cerevisiae transcriptome	S. cerevisiae DRS reads	0.77	0.88	0.82	25000	0.86	0.74	0.79	25000	0.82	0.81	0.80	50000

Table 3. DNN models trained on a transcriptome and tested on cDNA or direct RNA reads from the same species. Precision, recall (true positive rate), and F1-score for the models are given for each orientation separately, and for the total set. The number of reads tested are also provided. The human cDNA (1D) and DRS data corresponds to the JHU Run 1 available from https://github.com/nanopore-wgs-consortium/NA12878/blob/master/nanopore-human-transcriptome/fastq_fast5_bulk.md). The S. cerevisiae nanopore cDNA (SRR6059708) and DRS (SRR6059706) reads were obtained from SRA (SRP118556) (Garalde et al., 2018).

			Forward				Reverse				Average			
Model	Training	Testing	Prec.	Recall	F1	Reads	Prec.	Recall	F1	Reads	Prec.	Recall	F1	Reads
MLP	mouse transcriptome	human cDNA reads	0.60	0.94	0.73	21255	0.92	0.55	0.69	28745	0.79	0.71	0.71	50000
MLP	mouse transcriptome	Human DRS reads	0.88	0.86	0.87	24327	0.86	0.88	0.87	24326	0.87	0.87	0.87	48653
MLP	C. glabrata transcriptome	S. cerevisiae cDNA reads	0.96	0.93	0.94	23486	0.94	0.97	0.95	26514	0.95	0.95	0.95	50000
MLP	C. glabrata transcriptome	S. cerevisiae DRS reads	0.85	0.90	0.87	24844	0.90	0.84	0.87	24843	0.87	0.87	0.87	49687
MLP	Human transcriptome	S. cerevisiae DRS reads	0.77	0.79	0.78	24844	0.78	0.77	0.78	24843	0.78	0.78	0.78	49687
MLP	Human transcriptome	Sorghum PacBio reads	0.61	0.61	0.61	37239	0.61	0.62	0.62	37239	0.62	0.62	0.62	74478
CNN	Mouse transcriptome	human cDNA reads	0.79	0.90	0.84	21476	0.92	0.82	0.87	28525	0.85	0.86	0.85	50001
CNN	Mouse transcriptome	Human DRS reads	0.90	0.93	0.92	25000	0.93	0.90	0.91	25000	0.92	0.92	0.92	50000
CNN	C. glabrata transcriptome	S. cerevisiae cDNA reads	0.94	0.93	0.94	23095	0.94	0.95	0.95	26906	0.94	0.94	0.94	50001
CNN	C. glabrata transcriptome	S. cerevisiae DRS reads	0.86	0.89	0.88	25000	0.89	0.86	0.87	25000	0.88	0.88	0.88	50000

Table 4. Cross-species DNN models. Precision, recall (true positive rate), and F1-score for the model trained on the annotated transcriptome for mouse tested on human nanopore cDNA (1D JHU run 1) and direct RNA sequencing (DRS) reads (JHU, run 1) reads from (Workman et al., 2018) (https://github.com/nanopore-wgs-consortium/NA12878/blob/master/RNA.md). Orientation labels for cDNA reads were previously calculated by mapping them to the transcriptome (see Methods). The accuracy values for the reads oriented as obtained after mapping, and reverse-complemented from the mapping (Reverse), and the average value is provided. The number of reads used for testing is shown in the corresponding columns.. *S. cerevisiae* nanopore direct RNA reads were obtained from (Garalde et al., 2018) (https://trace.ncbi.nlm.nih.gov/Traces/sra/?study=SRP118556) and PacBio cDNA reads for Sorghum were obtained from (Abdel-Ghany et al., 2016) (https://zenodo.org/record/49944#.XCkXQC-ZN24).

Model	Cluster with >1 read	Clusters with >2 reads
Hs_MLP	81.21 %	87.68 %
Hs_CNN	83.91 %	89.29 %
Sc_MLP	85.82 %	88.11 %
Sc_CNN	85.43 %	92.85 %

Table 5. Clusters with more than 50% of reads correctly oriented. The table shows the percentage of clusters having more than 50% of the reads correctly oriented by ReorientExpress separated by species, human (Hs) or *S. cerevisiae* (Sc), and DNN model, MLP or CNN. The results are shown for clusters with more than 1 read or with more than 2 reads.

**Proportions**	**Hs_CNN**	**Hs_MLP**	**Sc_CNN**	**Sc_MLP**
**Default**	42.51 %	42.51 %	47.12 %	47.12 %
**ReorientExpress**	85.44 %	83.77 %	88.68 %	86.75 %
**ReorientExpress and clustering**	96.20 %	95.56 %	98.05 %	90.24 %
**Total Number of reads**	**Hs_CNN**	**Hs_MLP**	**Sc_CNN**	**Sc_MLP**
**Default**	114902	114902	1406529	1406529
**ReorientExpress**	230942	226425	2646930	2589360
**ReorientExpress and clustering**	260033	258289	2926643	2693432

Table 6. Reads correctly oriented. Proportions (upper table) and total number of reads correctly oriented from the Human (Hs) cDNA sample (270296 labelled reads) and the S. cerevisiae (Sc) cDNA sample (2984873 labelled reads).

			Forward				Reverse				Average			
Model	Training	Testing	Prec.	Recall	F1	Reads	Prec.	Recall	F1	Reads	Prec.	Recall	F1	Total Reads
MLP	Human cDNA reads	human transcriptome	0.85	0.81	0.83	25000	0.85	0.81	0.83	25000	0.83	0.83	0.83	50000
MLP	Human DRS reads	Human cDNA reads	0.67	0.70	0.68	20655	0.79	0.76	0.77	30450	0.74	0.74	0.74	51105
MLP	Human IVT RNA DRS reads	Human cDNA reads	0.75	0.61	0.67	21690	0.74	0.84	0.79	28320	0.74	0.72	0.73	50001
CNN	Human IVT RNA DRS reads	Human cDNA reads	0.80	0.72	0.75	21476	0.80	0.86	0.83	28525	0.80	0.79	0.79	50001
MLP	S. cerevisiae cDNA reads	S. cerevisiae transcriptome	0.87	0.88	0.88	3299	0.88	0.88	0.88	3299	0.88	0.88	0.88	6598

Table 7. Models trained on Nanopore reads. Precision (Prec.), recall (true positive rate), and F1-score for the model trained on cDNA or direct RNA nanopore reads from (Workman et al., 2018) (https://github.com/nanopore-wgs-consortium/NA12878/blob/master/RNA.md) for human, and tested on the human transcriptome; or trained on S. cerevisiae cDNA reads and tested on the *S. cerevisiae* transcriptome. The accuracy values for the reads oriented as obtained after mapping, and reverse-complemented from the mapping (Reverse), and the average value is provided. The number of reads used for testing is shown in the corresponding columns.

	Guppy - fast algorithm	Guppy - high accuracy
**Read length (bp)**	**% correctly predicted**	**% correctly predicted**
>50	54%	54%
>250	86%	87%
>500	92%	92%

Table 8. Testing the dependency with base-calling. Guppy fast and Guppy high accuracy with the signal files from the in-vitro transcript RNA sequenced with MinION by the Nanopore Consortium (available from https://github.com/nanopore-wgs-consortium/NA12878/blob/master/nanopore-human-transcriptome/fastq_fast5_bulk.md) are used. As this is direct RNA sequencing, the orientation of the reads can be used to test the accuracy of our models. The test was performed using 50,000 random IVT RNA reads with the MLP model trained on 50,000 random transcripts from human.

	Forward				Reverse				Average			
Trim	Precision	Recall	F1-score	Reads	Precision	Recall	F1-score	Reads	Precision	Recall	F1-score	Total reads
10nt	0.78	0.83	0.80	8468	0.88	0.85	0.86	12755	0.84	0.84	0.84	21223
20nt	0.78	0.83	0.80	8528	0.88	0.84	0.86	12892	0.84	0.84	0.84	21420
40nt	0.77	0.82	0.80	8531	0.88	0.84	0.86	12916	0.83	0.83	0.83	21447
80nt	0.76	0.82	0.79	8518	0.88	0.83	0.85	12855	0.83	0.83	0.83	21373
100nt	0.75	0.82	0.78	8521	0.86	0.83	0.84	12957	0.81	0.82	0.82	21478
200nt	0.74	0.80	0.77	8418	0.82	0.80	0.81	12825	0.79	0.80	0.79	21253

Table 9. Testing on trimmed reads. The human model (trained on the transcriptome) and tested on the human cDNA reads after trimming a certain number of nucleotides from both ends of the read: 10, 20, 40, 80, 100, and 200. In each case, the accuracy values are calculated as before.

	Forward				Reverse				Average			
Trim	Precision	Recall	F1-score	Reads	Precision	Recall	F1-score	Reads	Precision	Recall	F1-score	Reads
0	0.71	0.81	0.76	5139	0.84	0.76	0.80	6898	0.79	0.78	0.78	12037
50	0.73	0.80	0.76	5136	0.84	0.78	0.81	7158	0.79	0.79	0.79	12474
100	0.72	0.79	0.75	5359	0.83	0.78	0.80	7241	0.79	0.78	0.78	12600
150	0.69	0.79	0.74	5329	0.82	0.74	0.78	7184	0.77	0.76	0.76	12513
200	0.67	0.81	0.73	5170	0.83	0.70	0.76	6924	0.76	0.75	0.75	12094

Table 10. Training with trimmed transcripts. The MLP human models were trained on the transcriptome after trimming the corresponding nucleotides from both ends. The test was performed on the human cDNA reads without any trimming.

			Forward				Reverse				Average			
Model	Training	Testing	Prec.	Recall	F1	Reads	Prec.	Recall	F1	Reads	Prec.	Recall	F1	Total Reads
Random Forest	Human transcriptome	human cDNA reads	0.65	0.69	0.67	21255	0.76	0.72	0.74	28745	0.71	0.71	0.71	50000
Random Forest	S. cerevisiae transcriptome	S. cerevisiae cDNA reads	0.87	0.82	0.85	23486	0.85	0.89	0.87	26514	0.86	0.86	0.86	50000
SVM	Human transcriptome	human cDNA reads	0.71	0.69	0.70	21255	0.78	0.79	0.78	28745	0.75	0.75	0.75	50000
SVM	S. cerevisiae transcriptome	S. cerevisiae cDNA reads	0.96	0.93	0.95	23486	0.94	0.97	0.95	28745	0.95	0.95	0.95	50000

Table 11. Accuracy of Random Forest and SVM models. Precision (Prec.), recall (true positive rate), and F1-score (F1) for each model based on k-mers k=1,…,5, trained on the annotated human or S. cerevisiae transcriptomes and tested on nanopore cDNA reads from the same species (JHU, 1D cDNA reads, run 1, from NA12878 for human https://github.com/nanopore-wgs-consortium/NA12878/blob/master/nanopore-human-transcriptome/fastq_fast5_bulk.md, and SRR6059708 for S. cerevisiae). For the Random Forest the default parameters were used except for the maximum depth, which was tested from 1 to 10. The best accuracy was obtained with depth 9, which is the one reported. The complete list of parameters used for training the random forest and SVM is provided below.

RandomForestClassifier(bootstrap=True, class_weight=None, criterion='gini', max_depth=9, max_features='auto', max_leaf_nodes=None, min_impurity_decrease=0.0, min_impurity_split=None, min_samples_leaf=1, min_samples_split=2, min_weight_fraction_leaf=0.0, n_estimators=10, n_jobs=1, oob_score=False, random_state=0, verbose=0, warm_start=False).

For the SVM different kernels: rbf, linear and poly were tested. The best accuracy model parameters are below:

SVC(C=1.0, cache_size=200, class_weight=None, coef0=0.0, decision_function_shape='ovr', degree=3, gamma='auto', kernel='linear', max_iter=-1, probability=False, random_state=None, shrinking=True, tol=0.001, verbose=False).

Incidentally, the SVM was much slower than RFs. For comparison: for the human dataset and using the same computer, the RF took 421 seconds (around 7 min) but the SVM took 47825 seconds (>13 hours), i.e. 100 times slower.

	Forward				Reverse				Average			
	Prec.	Recall	F1	Reads	Prec.	Recall	F1	Reads	Prec.	Recall	F1	Reads
Sorghum model tested on Sorghum Pacbio	0.95	0.95	0.95	25000	0.95	0.95	0.95	25000	0.95	0.95	0.95	50000
Maize model tested on Sorghum Pacbio	0.94	0.95	0.95	25000	0.95	0.94	0.95	25000	0.95	0.95	0.95	50000

Table 12. Testing of ReorientExpress with PacBio data. Precision, recall (true positive rate), and F1-score for the model trained on the annotated transcriptome for Sorghum (Sorghum bicolor NCBIv3) and Maize (Zea Mays B73_RefGen_v4) tested on PacBio cDNA reads for Sorghum (Abdel-Ghany et al., 2016) (https://zenodo.org/record/49944#.XCkXQC-ZN24). The accuracy values for the reads oriented as obtained after mapping, and reverse-complemented from the FASTA/FASTQ, and the average value is provided. The number of reads used for testing is shown in the corresponding columns.

References

Abdel-Ghany, S.E., Hamilton, M., Jacobi, J.L., Ngam, P., Devitt, N., Schilkey, F., Ben-Hur, A., and Reddy, A.S.N. (2016). A survey of the sorghum transcriptome using single-molecule long reads. Nat. Commun. *7*, 11706.

Garalde, D.R., Snell, E.A., Jachimowicz, D., Sipos, B., Lloyd, J.H., Bruce, M., Pantic, N., Admassu, T., James, P., Warland, A., et al. (2018). Highly parallel direct RNA sequencing on an array of nanopores. Nat. Methods *15*, 201–206.

Workman, R.E., Tang, A., Tang, P.S., Jain, M., Tyson, J.R., Zuzarte, P.C., Gilpatrick, T., Razaghi, R., Quick, J., Sadowski, N., et al. (2018). Nanopore native RNA sequencing of a human poly(A) transcriptome. BioRxiv.
