# Supplementary material for: ReorientExpress: reference-free orientation of nanopore cDNA reads with deep learning
Source: Genome Biol. 2019 Nov 29;20:260. doi: 10.1186/s13059-019-1884-z (PMC6883653; doi:10.1186/s13059-019-1884-z)
Supplement: Supplementary file 4 — Additional file 4. Review history. [file 13059_2019_1884_MOESM4_ESM.docx]

Review History

**First round of review**

**Reviewer 1**

**Were you able to assess all statistics in the manuscript, including the appropriateness of statistical tests used?**

Yes: Insufficient details were provided for me to make good judgement.

**Were you able to directly test the methods?**

No

**Comments to author:**

In this article, Ruiz-Reche et al. propose a machine learning method that can predict the orientation of an RNA read solely based on the nucleotides. The method seems to achieve high accuracy in doing that. However, it's not clear if these results are solid based on the presentation of the work. I also have concerns about the design of the deep learning model and other machine learning methods used here.

About the deep learning method and the comparing Markov model:

*	The authors developed a multi-layer perceptron (MLP) for RNA sequences, which seems to be antique. For sequence data, convolutional neural net (CNN) and recurrent neural net (RNN) are more suitable than MLP. What's more, MLP requires the input to have fixed size. How do you deal with RNA sequences of variable lengths? There is no mention of this in the article.

*	The biological motivation is that the orientation can be detected based on motifs that exist in RNA sequences. However, the authors didn't demonstrate that their method can detect those motifs.

*	Why do you use normalized k-mer counts as features? What's the motivation behind that? Why not use RNA sequences and one-hot encoding directly?

*	The description of Markov model is very brief and vague. Is this a hidden Markov model or other types of Markov models? It's unclear to me how the Markov model was trained on the sequence data.

*	The choice of Markov model as a comparing method is strange and suspicious. Maybe this problem is too easy for deep learning. Why not use a machine learning method that requires less parameter tuning, such as logistic regression, random forest and support vector machines?

Overall, the presentation of the work is not sufficient for readers to understand what the authors did and to assess its scientific merits.

*	The calculation of normalized k-mer counts seems to be essential to the success of the method. However, there is only one sentence to describe how this was done. It's very hard for me to understand how the k-mers were counted and constructed as input for the MLP. It's better to draw a figure to illustrate this process.

*	There is no mentioning how large the datasets are in the results section. It's hard for me to judge whether the 0.75-0.125-0.125 split is reasonable or not.

*	Where is the dropout layer inserted? It's better to draw a figure to show your deep learning network.

*	Why do you randomly select half the sequences and reverse complement them? Why not simply reverse complement all the sequences, if you want to make the dataset label balanced?

*	On page 4, line 3, what's RNP? This is not previously defined.

*	Figure 1's legend is not even attached to the figure itself.

*	On page 9, line 2, I believe it's mm9, not mm19.

*	On page 6, line 22, "ReorientExpress predicted correctly more than 50% of the reads in a cluster". Why is this significant? A random guess can give you 50% accuracy, right?

Some additional comments:

*	The authors present the method as if it works only with Oxford nanopore reads. It seems the method can work with any RNA reads. What about other types of reads?

*	Please use standard English. The article does not read smoothly.

===

Please consider the following questions:

- Are the methods appropriate to the aims of the study, are they well described, and are necessary controls included?

- Are the conclusions adequately supported by the data shown?

- Are sufficient details provided to allow replication and comparison with related analyses that may have been performed?

- Is the method likely to be of broad utility? Is any software component easy to install and use? Please indicate briefly the novel features and/or advantages of the method, and/or please reference the relevant publications and which methods, if any, it should be compared with.

- Is the paper of broad interest to others in the field, or of outstanding interest to a broad audience of biologists?

**Reviewer 2**

**Were you able to assess all statistics in the manuscript, including the appropriateness of statistical tests used?**

Yes: The statistical methodology is generally sound.

**Were you able to directly test the methods?**

No

**Comments to author:**

The authors present a computational model to classify the orientation of ONT long read RNA sequencing reads. Albeit the method seems to perform well enough for this task, there are a few technical issues that need to be addressed. In particular, the claims that the method leverages features of the strand-specific RNP code to make the predictions should be further substantiated with additional testing (see specific comments below) and biological validation. A broader concern lies in the significance of the method. Indeed, the rationale underlying the importance of proper transcript orientation should be expanded, and a comparative evaluation of the efficiency of strand assignment using alternative methods (such as those the authors list in the introduction) is essentially to reinforce the pertinence and significance of ReorientExpress. For instance, the authors mention that matching stranded cDNA adapters is a problem given the error rate, but there is no reference or data to support this. Given the latest single molecule base calling accuracies with ONT's guppy 'flip flip' algorithm (~94%), the size of stranded cDNA adapters (>20nt), and their different sequence composition, it should be straightforward to match them using sequence similarity alone. This is much a more intuitive method than relying on obtuse deep learning, although both methods are complementary. Nonetheless, the authors should include this classical approach in their comparison and evaluation of ReorientExpress. The github repository is well detailed and the code seems to work without significant bugs.

-How does the method compare to other tools, such as those developed specifically for this purpose, i.e. https://github.com/nanoporetech/pychopper

-Is the method biased for poly-A / RNApol2 transcripts? Can ReorientExpress accurately classify cDNA isoforms derived from random hexamer priming?

-How do the models trained in reorientExpress perform on PacBio data?

-How long does it take to train and execute on typical MinION output?

-DRS data requires the 3' end of the RNA polymer to be ligated to the sequencing adapter, thus having the potential to contain much longer poly-A tails than cDNA reads, which can be primed internally. The authors test the effect of truncating the cDNA during testing, but what about truncating the training set? this should provide more thorough support to the authors' claims that internal motifs of the 'RNP code' are leveraged by the DNN to classify the orientation of reads.

-Similarly, what happens if the DNN is trained with cDNA data and tested on dRNA data? Or with reference transcripts (i.e. Gencode)?

-How is the inter-species accuracy: for instance, is the human model able to predict the orientation of mouse reads? Do users need to train a model each time for a species of interest (on the same or related species)?

-when using only primary mappings, is the MAPQ also used? What about primary mapping with supplementary or secondary alignments with same (similar) scores?

-How does the base calling affect prediction? The authors should compare 2 different versions of ONT Guppy base caller (high accuracy and rapid) to assess the impact of base callers, which are constantly updated, on the accuracy of ReorientExpress.

-The authors need to demonstrate that their method is appropriate for antisense RNAs which share significant exonic overlap in the genome.

- look at additional files.

- loss in accuracy could also be explained by RNA modifications present in the DRS data and not the cDNA data. (check source of cDNA).

- Not clear why a model with all [1-5]-mers is used. Why not just K=5? What is the impact of k-mer size on accuracy?

-(check reference #9)

-Are there any systematic features of reads that do not get classified properly (nucleotide content, splicing levels, ncRNA vs mRNA)? Are biological properties of the transcripts important for the classification of their orientation?

-The section on clustering could use clarification. For instance, how many reads in total are in clusters where the majority vote is wrong? Are there any clusters that contain antisense (lncRNA) transcripts in addition to 'sense' transcripts?

**Authors Response**

**Point-by-point responses to the reviewers’ comments:**

Reviewer #1:

In this article, Ruiz-Reche et al. propose a machine learning method that can predict the orientation of an RNA read solely based on the nucleotides. The method seems to achieve high accuracy in doing that. However, it's not clear if these results are solid based on the presentation of the work. I also have concerns about the design of the deep learning model and other machine learning methods used here.

About the deep learning method and the comparing Markov model:

* The authors developed a multi-layer perceptron (MLP) for RNA sequences, which seems to be antique. For sequence data, convolutional neural net (CNN) and recurrent neural net (RNN) are more suitable than MLP. What's more, MLP requires the input to have fixed size. How do you deal with RNA sequences of variable lengths? There is no mention of this in the article.

*Answer:*

*ReorientExpress originally implements a deep neural network (DNN) as a multi-layer percentron (MLP) with several inner layers (described in Additional file1: Table 1). We used as input the normalized count of k-mers (k=1,...,5) in each transcript molecule to ensure that the input is of fixed size for transcripts with different lengths. Thus our MLP does work on variable length transcripts and the input sequences are transformed into a fixed-length input. We have indicated this explicitly in the text.*

*Although MLPs are simpler than Convolutional (CNNs) and Recurrent (RNNs) Neuronal Networks, MLPs are faster to train and to run. Also, the inner layers of an MLP exploit the non-linear power of neural networks in a simpler setting. CNNs and RNNs indeed present some advantages, as CNNs can model relative spatial relationships through filters and RNNs can process multiple sequential inputs.*

*For their ability to model sequence context, we trained and tested a CNN using the same datasets, which is now included in ReorientExpress (described in Figure 1 and Additional file 1: Table 2). The CNN is implemented on sequence windows of 500nt with one hot encoding. The orientation of a read is decided by calculating for each orientation, forward or reverse, the mean of the probabilities for all windows, and selecting the highest one. We have added in the text the details of this CNN and the benchmarking results. There is a marginal gain in the CNN performance compared to MLP.*

Reviewer #1:

* The biological motivation is that the orientation can be detected based on motifs that exist in RNA sequences. However, the authors didn't demonstrate that their method can detect those motifs.

*Answer:*

*We used the active features from the first layer of the CNN to obtain the sequences that are most informative for predicting the orientation, using an approach similar to PMID: 26213851 and PMID: 29970003. To explore exhaustively all potential RNA motifs, we extracted the 32 filters that showed activations above 0 and converted the associated sequences to position weight matrices (PWMs). We then compared the derived matrices with the CISBP-RNA database (http://cisbp-rna.ccbr.utoronto.ca/) using the TOMTOM algorithm for the comparison of PWM-based motifs (http://meme-suite.org/doc/tomtom.html). From this analysis we obtained a number of filters with significant similarity to known motifs for RNA-protein binding. We have included these results in Figure 3 and in Additional files 2 and 3. These analyses have now been added to the modified version of the manuscript.*

Reviewer #1:

* Why do you use normalized k-mer counts as features? What's the motivation behind that? Why not use RNA sequences and one-hot encoding directly?

*Answer:*

*We hypothesized that the sequence properties that would capture the orientation of the RNA would be best represented as short motifs. We therefore used all k-mers from k=1 to k=5. Other maximum k-mer sizes are possible (included as an option in the software). Additionally, short motifs would account for the fact that base-call errors may not allow to capture correctly longer sequence motifs. The use of k-mers also allowed us to have a fixed-length input from a variable length sequence. Furthermore, neural networks generally work better with input values between 0 and 1, as the k-mer densities used; We have added this description in the Methods section. We implemented the direct one-hot encoding from the nucleotide sequence on the CNN model, which is now included in the manuscript (described in Figure 1 and in Methods).*

*Considering one-hot encoding as a generic way to represent categorical data for Machine Learning algorithms that need numerical data as input, we also tried one-hot encoding of the k-mer sets in the MLP. That is, we recorded a 1 if a k-mer was present, and 0 otherwise. That is, given sequence S and k-mer s, h(s) = 1, if S contains s, 0 otherwise. For instance, for “ATTG”:*

*(h(A), h(C), h(G), h(T), h(AA), h(AT), h(AG),....) = (1,0,1,1,0,1,0,...). The one-hot encoding on the k-mers in the MLP performed slightly worse than the model based on the k-mer frequencies: average precision and recall = 0.71.*

Reviewer #1:

* The description of Markov model is very brief and vague. Is this a hidden Markov model or other types of Markov models? It's unclear to me how the Markov model was trained on the sequence data.

*Answer:*

*This was done simply to test a different way of using the k-mer frequencies for prediction that did not take into account their co-occurrences in transcripts. We have now added comparisons to an SVM and a Random Forest, which performe better than the Markov model.*

Reviewer #1:

* The choice of Markov model as a comparing method is strange and suspicious. Maybe this problem is too easy for deep learning. Why not use a machine learning method that requires less parameter tuning, such as logistic regression, random forest and support vector machines?

*Answer:*

*There was no intention to mislead by using a Markov model for comparison. Markov models can be quite effective to identity sequence motifs related to RNA binding proteins or the coding potential of an RNA. That is why we chose this approach for comparison. We have now trained and tested a support vector machine (SVM) and a Random Forest (RF) with the same datasets, using as inputs the same k-mer frequencies. Both show worse accuracy than the MLP and CNN models. These results are given in Additional file 1: Table 11, and are discussed in the manuscript.*

Reviewer #1:

Overall, the presentation of the work is not sufficient for readers to understand what the authors did and to assess its scientific merits.

* The calculation of normalized k-mer counts seems to be essential to the success of the method. However, there is only one sentence to describe how this was done. It's very hard for me to understand how the k-mers were counted and constructed as input for the MLP. It's better to draw a figure to illustrate this process.

*Answer:*

*We have included extra information on how the normalized k-mer counts are calculated.*

*The frequency was calculated as follows: freq(k-mer) = count(k-mer) / (L-k+1), where L is the length of the input transcript, k is the size of the k-mer, and “k-mer” indicates a specific word of length k. That is, the denominator is the number of possible k-mers in a sequence of length L. The input is thus a vector with the frequencies for all k-mers from k=1, to k selected as parameter (we used 5 in the paper).*

Reviewer #1:

* There is no mentioning how large the datasets are in the results section. It's hard for me to judge whether the 0.75-0.125-0.125 split is reasonable or not.

*Answer:*

*Apologies for this oversight, we had only included this information for the analysis of the yeasts. The information on the size of the datasets is now provided in the methods section: We trained the models using 50000 randomly selected transcript sequences from the annotation. The ROC curves and the accuracy related to the clustering and transcript types, now described in Figures 2 and 3, used the prediction over all cDNA reads. The accuracy tables provided in Additional file 1 are generally calculated on a set of 50000 random reads, unless otherwise stated.*

Reviewer #1:

* Where is the dropout layer inserted? It's better to draw a figure to show your deep learning network.

*Answer:*

*We provide now in Additional file 1: Table 1 the description of the MLP and in Additional file 1: Table 2 the description of the CNN. The MLP has 5 dense layers, and we inserted a droup-out layer after each dense layer, except for the last dense layer, after which we placed the output with a sigmoid function. The CNN model consists of 3 convolutional layers, 3 pooling layers and 3 dense layers, with different filter sizes.*

Reviewer #1:

* Why do you randomly select half the sequences and reverse complement them? Why not simply reverse complement all the sequences, if you want to make the dataset label balanced?

*Answer:*

*We used this approach to have two independent datasets in terms of their sequence content. We have also performed the analysis by reverse-complementing all sequences. This improves the accuracy slightly, so we have added this as an option in the command line. For instance, for the CNN model the average precision improves from 0.84 to 0.88, and the average recall from 0.85 to 0.88.*

Reviewer #1:

* On page 4, line 3, what's RNP? This is not previously defined.

*Answer:*

*Apologies for the oversight. We have modified the text to make it more clear: ReorientExpress approach builds on the hypothesis that RNA molecules present sequence biases and motifs relevant to their metabolism, generally related to protein binding sites...*

Reviewer #1:

* Figure 1's legend is not even attached to the figure itself.

*Answer:*

*This formatting issue has been corrected.*

Reviewer #1:

* On page 9, line 2, I believe it's mm9, not mm19.

*Answer:*

*Thanks for pointing this out. We have corrected this error. We now refer to Genocode annotation M19 (https://www.gencodegenes.org/mouse/release_M19.html).*

Reviewer #1:

* On page 6, line 22, "ReorientExpress predicted correctly more than 50% of the reads in a cluster". Why is this significant? A random guess can give you 50% accuracy, right?

*Answer:*

*The complete sentence says “For the majority of clusters with >2 reads … ReorientExpress predicted correctly more than 50% of the reads in a cluster”. The significance lies in the fact that this happens for the majority of clusters. A random guess would give that approximately 50% of clusters have more than 50% of their reads correctly oriented. However, we found that more than 80% of clusters have more than 50% of their reads correctly oriented. The implication is that a majority vote in those 80% of clusters would label all reads in the cluster correctly. This happens for the majority of clusters in human and yeast, which in turns is reflected in an increase in the number of correctly oriented reads. The numbers are now given in Tables 5 and 6 from Additional file 1, and the results are shown in Figure 2. We have also tried to clarify this in the text.*

Reviewer #1:

Some additional comments:

* The authors present the method as if it works only with Oxford nanopore reads. It seems the method can work with any RNA reads. What about other types of reads?

*Answer:*

*We had already tested the MLP model (trained on the human transcriptome) on PacBio cDNA reads from Sorghum (http://www.ncbi.nlm.nih.gov/pubmed/27339290). This was included in the original manuscript in Additional file 1, Table 13. We have now trained the model using the Sorghum and Maize annotations from Ensembl (cDNA annotation files) for Zea Mays B73_RefGen_v4 http://plants.ensembl.org/Zea_mays/Info/Index and for Sorghum bicolor Sorghum_bicolor_NCBIv3 http://plants.ensembl.org/Sorghum_bicolor/Info/Index. The results are now included in Additional file 1: Table 12, and they are described in the article.*

Reviewer #1:

* Please use standard English. The article does not read smoothly.

*Answer:*

*We have proofread the article and tried to improve the use of English.*

Reviewer #2:

The authors present a computational model to classify the orientation of ONT long read RNA sequencing reads. Albeit the method seems to perform well enough for this task, there are a few technical issues that need to be addressed. In particular, the claims that the method leverages features of the strand-specific RNP code to make the predictions should be further substantiated with additional testing (see specific comments below) and biological validation. A broader concern lies in the significance of the method. Indeed, the rationale underlying the importance of proper transcript orientation should be expanded, and a comparative evaluation of the efficiency of strand assignment using alternative methods (such as those the authors list in the introduction) is essentially to reinforce the pertinence and significance of ReorientExpress. For instance, the authors mention that matching stranded cDNA adapters is a problem given the error rate, but there is no reference or data to

support this. Given the latest single molecule base calling accuracies with ONT's guppy 'flip flip' algorithm (~94%), the size of stranded cDNA adapters (>20nt), and their different sequence composition, it should be straightforward to match them using sequence similarity alone. This is much a more intuitive method than relying on obtuse deep learning, although both methods are complementary. Nonetheless, the authors should include this classical approach in their comparison and evaluation of ReorientExpress. The github repository is well detailed and the code seems to work without significant bugs.

-How does the method compare to other tools, such as those developed specifically for this purpose, i.e. https://github.com/nanoporetech/pychopper

*Answer:*

*We run pychopper (https://github.com/nanoporetech/pychopper) on all cDNA reads that we mapped previously to transcripts to label their orientation (270296 human cDNA reads uniquely mapped to annotated transcripts with MAPQ = 60). Pychopper made predictions for only 24% of the reads, from which 98% were correctly classified. So in total, from the 270296 reads tested, pychopper could classify accurately ~23.5% (~63520 reads). All reads had been recalled with Guppy (https://github.com/nanopore-wgs-consortium/NA12878).*

Reviewer #2:

Is the method biased for poly-A / RNApol2 transcripts?

*Answer:*

*All models were tested on Poly-A+ long-read sequencing reads. Nonetheless, we tested whether there were subtypes of RNAs that were better predicted than others. We annotated cDNA reads by transcript type by mapping them to the transcriptome annotation (Gencode v27) with minimap2, selecting only reads mapped with at least MAPQ 60, and keeping only uniquely mapped reads. We then tested the proportion of each type that was correctly predicted. This result is shown in Figure 3 and described in the text.*

Reviewer #2:

Can ReorientExpress accurately classify cDNA isoforms derived from random hexamer priming?

*Answer:*

*The methods developed are based on the sequence content of the reads and are robust to variable read lengths as well as to the truncation of cDNA reads, and of the training data, as shown in Tables 9 and 10 from the Additional file 1. We expect the DNN models will work also on cDNA isoforms derived from random hexamer priming.*

Reviewer #2:

-How do the models trained in reorientExpress perform on PacBio data?

*Answer:*

*We had already tested the MLP model (trained on the human transcriptome) on PacBio cDNA reads from Sorghum (http://www.ncbi.nlm.nih.gov/pubmed/27339290). This was included in the original manuscript in Additional file 1, Table 13. We have now retrained the model using the Sorghum and Maize annotations from Ensembl: Zea Mays - B73_RefGen_v4 http://plants.ensembl.org/Zea_mays/Info/Index, and Sorghum bicolor - Sorghum_bicolor_NCBIv3 http://plants.ensembl.org/Sorghum_bicolor/Info/Index*

*and have tested the models on the same PacBio reads used before (from http://www.ncbi.nlm.nih.gov/pubmed/27339290). The results are now included in Additional file 1: Table 12, and they are described in the article.*

Reviewer #2:

-How long does it take to train and execute on typical MinION output?

*Answer:*

*Training is best performed on the annotation from the same or a related species and will depend on the size of the input. The largest part is spent in data processing. Training the MLP with 50,000 random transcripts from the human annotation takes 19.7 minutes (15.4 with the mouse annotation), whereas training with the full S. cerevisiae annotation takes 1.2 minutes (1.0 minute with C. glabrata annotation). Testing on 50,000 random cDNA reads takes between 4.6 and 4.9 minutes.*

Reviewer #2:

-DRS data requires the 3' end of the RNA polymer to be ligated to the sequencing adapter, thus having the potential to contain much longer poly-A tails than cDNA reads, which can be primed internally. The authors test the effect of truncating the cDNA during testing, but what about truncating the training set? this should provide more thorough support to the authors' claims that internal motifs of the 'RNP code' are leveraged by the DNN to classify the orientation of reads.

*Answer:*

*We truncated the training set by different lengths and observed similar accuracies despite the trimming. The implication of this is that using partial or fragmented annotations for training does not impact accuracy significantly. This is shown in Additional file 1: Table 10. On the other hand, as we showed in the original manuscript, truncating reads still maintain a reasonably high accuracy, which is relevant for the application to reads that might be fragmented due to internal priming. This is shown in Additional file 1: Table 9.*

Reviewer #2:

-Similarly, what happens if the DNN is trained with cDNA data and tested on dRNA data? Or with reference transcripts (i.e. Gencode)?

*Answer:*

*We trained DNN models with the cDNA reads and tested them on the annotated transcriptomes from the same species. The accuracy is almost as high as with the model trained on transcriptomes and tested on cDNA reads. However, the human model trained on direct RNA sequencing reads and tested on cDNA data yielded much lower accuracy. These results are shown in Additional file 1, Table 7.*

Reviewer #2:

-How is the inter-species accuracy: for instance, is the human model able to predict the orientation of mouse reads? Do users need to train a model each time for a species of interest (on the same or related species)?

*Answer:*

*We showed that the models also work across species: The mouse model is able to predict the orientation of human reads. The model would only need to be trained once. The same model trained in one species could be applied to samples from the same or related species. For instance, users could use a C. glabrata model to make predictions for other yeast species, as we show in the article. The aim was to show that one can train on a related species and apply ReorientExpress on a different species that does not have an annotation available.*

Reviewer #2:

-when using only primary mappings, is the MAPQ also used? What about primary mapping with supplementary or secondary alignments with same (similar) scores?

*Answer:*

*We used mappings with maximum mapping quality (MAPQ = 60). We further only kept uniquely mapping reads, i.e. we removed the ~3% of reads that were multimapping. We worked with the remaining cDNA reads. This is now described in more detail in the methods section.*

Reviewer #2:

-How does the base calling affect prediction? The authors should compare 2 different versions of ONT Guppy base caller (high accuracy and rapid) to assess the impact of base callers, which are constantly updated, on the accuracy of ReorientExpress.

*Answer:*

*We used Guppy rapid and Guppy high accuracy with the signal files from the in-vitro transcript RNA sequenced with MinION by the Nanopore Consortium (available from https://github.com/nanopore-wgs-consortium/NA12878/blob/master/nanopore-human-transcriptome/fastq_fast5_bulk.md ). As this is direct RNA sequencing, we can use the orientation of the reads to test the accuracy of our models. We performed the test using 50000 IVT RNA reads using the human-transcriptome model. We did not observe major differences between the two base-called reads. This is now shown in Additional file 1: Table 8, and discussed in the Results section.*

Reviewer #2:

-The authors need to demonstrate that their method is appropriate for antisense RNAs which share significant exonic overlap in the genome.- look at additional files.

*Answer:*

*We annotated cDNA reads for their orientation and transcript type by mapping them to the transcriptome annotation (Gencode v27) with minimap2 (unspliced mapping), selecting only reads mapped with maximum quality (MAPQ=60), and keeping only uniquely mapped reads. We then tested the proportion of each type that was correctly predicted within each transcript type. Reads assigned to Antisense and TEC (To be Experimentally Confirmed) were correctly predicted for 60-70% of cases. All other types were predicted correctly for more than 70% of cases. This is now shown in Figure 3.*

Reviewer #2:

- loss in accuracy could also be explained by RNA modifications present in the DRS data and not the cDNA data. (check source of cDNA).

*Answer:*

*Indeed, we observed a lower accuracy when using DRS for training and cDNA for testing. This could be due to RNA modifications being present as sequence errors, and therefore hindering the construction of a model that is generalizable to cDNA reads. We have added a comment regarding this. In relation to this, we also tested the MLP model on in-vitro transcript RNA sequenced with the DRS protocol ( available from https://github.com/nanopore-wgs-consortium/NA12878/blob/master/nanopore-human-transcriptome/fastq_fast5_bulk.md) and the accuracy was much higher; hence, further indicating that current base-callers with DRS reads hinder the identification of motifs due to the presence of biochemically modified bases.*

Reviewer #2:

- Not clear why a model with all [1-5]-mers is used. Why not just K=5? What is the impact of k-mer size on accuracy? -(check reference #9)

*Answer:*

*We tested different models using fixed k. We observed that using k=1,...,5 improved slightly from using a fixed k. For k=1, average precision and recall = 0.68; for k=2, average precision and recall = 0.76; for k=3, average and recall = 0.79; for k=4, average precision and recall = 0.81; for k=5, average precision and recall = 0.80.*

Reviewer #2:

-Are there any systematic features of reads that do not get classified properly (nucleotide content, splicing levels, ncRNA vs mRNA)? Are biological properties of the transcripts important for the classification of their orientation?

*Answer:*

*We looked at two properties: length of the reads and read type. For the lengths of the reads, we observed that shorter reads are predicted with worse accuracy. We performed the test using 50000 random direct-RNA reads from in-vitro transcribed (IVT) RNA using the human-transcriptome model. Allowing shorter read lengths the MLP model showed worse accuracy, whereas the accuracy increased when restricting the prediction to longer reads. This is now shown in Additional file 1, Table 8. For read types, we observed that reads that we could assigned with some confidence to antisense transcripts, are less accurately predicted compared with other read types. Reads assigned to protein coding transcripts are the ones predicted with highest accuracy. Long non-coding RNAs are also predicted with higher accuracy than antisense reads. This is now described in the text and shown in Figure 3.*

Reviewer #2:

-The section on clustering could use clarification. For instance, how many reads in total are in clusters where the majority vote is wrong?

*Answer:*

*We have added more information about the clustering analysis in the paper. We have included in Additional file 1: Table 5 the proportion of clusters that have more than 50% of their reads correctly oriented. We have also included in Additional file 1: Table 6 the proportion of all cDNA reads in human (270296) or in S. cerevisiae (2984873) that are correctly oriented after using the majority vote in clusters. The number of reads that would be in clusters where the majority is wrong would be the difference to complete 100% of reads. This is as small as 1.95% (~58205 reads) for the CNN model in S. cerevisiae, or as 3.80% (~10271) for the CNN model in human.*

Reviewer #2:

Are there any clusters that contain antisense (lncRNA) transcripts in addition to 'sense' transcripts?

*Answer:*

*Using our labelling of cDNA reads through the mapping to annotated transcripts, and using the clustering results, we looked at all the clusters that contained 1 or more reads of type antisense. We then plotted the distribution of clusters according to the proportion of reads in each cluster that were antisense. Most of the clusters analysed had more than 90% of their reads of type antisense. This is shown in Additional file 1: Figure 2.*

*Some clusters still include antisense in a low proportion (<50%), which could explain why reads from antisense reads are harder to predicted correctly than other reads. However, the plot shows that the majority of clusters with antisense reads are quite “pure”: The clusters that have 100% of their reads labeled as antisense are 201 (78%), which correspond to 882 antisense reads from the total of 976 antisense reads, i.e. 90%. This is now shown as Additional file 1: Figure 2.*

**Second round of review**

**Reviewer 3**

I have now read through the paper and reviewer comments. I agree with your assessment that the authors have done addressed all of the major comments and concerns from both reviewers. The details of their deep learning framework, while dense, are sufficient for an expert who may want to use this work (and appropriately placed in supporting files). I also liked that they describe the structure of the various networks that they used.

I will say that while the writing is not bad, it is a bit dense and could use simplification for a general audience. This is more of an editorial decision. Another thing that I felt could be improved is additional text and emphasis on how this work can help different types of biological analyses.
